# Fasciclin-like arabinogalactan gene family in *Nicotiana benthamiana*: genome-wide identification, classification and expression in response to pathogens

**DOI:** 10.1186/s12870-020-02501-5

**Published:** 2020-07-01

**Authors:** Xinyang Wu, Yuchao Lai, Lanqing Lv, Mengfei Ji, Kelei Han, Dankan Yan, Yuwen Lu, Jiejun Peng, Shaofei Rao, Fei Yan, Hongying Zheng, Jianping Chen

**Affiliations:** 1 College of Agriculture and Biotechnology, Zhejiang University, Hangzhou, 310058 China; 2 State Key Laboratory for Managing Biotic and Chemical Threats to the Quality and Safety of Agro-products, Key Laboratory of Biotechnology in Plant Protection of Ministry of Agriculture and Zhejiang Province, Institute of Plant Virology, Ningbo University, Ningbo, 315211 China

**Keywords:** *N. benthamiana*, Fasciclin-like arabinogalactan proteins, Gene expression, Abiotic stress, Turnip mosaic virus

## Abstract

**Background:**

*Nicotiana benthamiana* is widely used as a model plant to study plant-pathogen interactions. Fasciclin-like arabinogalactan proteins (FLAs), a subclass of arabinogalactan proteins (AGPs), participate in mediating plant growth, development and response to abiotic stress. However, the members of FLAs in *N. benthamiana* and their response to plant pathogens are unknown.

**Results:**

38 *NbFLAs* were identified from a genome-wide study. *NbFLAs* could be divided into four subclasses, and their gene structure and motif composition were conserved in each subclass. *NbFLAs* may be regulated by cis-acting elements such as STRE and MBS, and may be the targets of transcription factors like C2H2. Quantitative real time polymerase chain reaction (RT-qPCR) results showed that selected *NbFLAs* were differentially expressed in different tissues. All of the selected *NbFLAs* were significantly downregulated following infection by turnip mosaic virus (TuMV) and most of them also by *Pseudomonas syringae pv tomato* strain DC3000 (*Pst* DC3000), suggesting possible roles in response to pathogenic infection.

**Conclusions:**

This study systematically identified *FLAs* in *N. benthamiana*, and indicates their potential roles in response to biotic stress. The identification of *NbFLAs* will facilitate further studies of their role in plant immunity in *N. benthamiana.*

## Background

The plant cell wall is a dynamic and complex organelle, which is mainly composed of cellulose, hemicellulose, pectins, glycans and proteins. It is not only involved in mechanical protection and structural support, but also in signal transduction, intercellular communication and immunity [1–3].

Hydroxyproline-rich glycoproteins (HRGPs) are typical cell-wall proteins that participate in plant growth, development and immunity [4, 5]. HRGPs have a few repetitive glycosylation motifs containing hydroxyproline (Hyp) residues that are glycosylation sites. Based on the different levels of O-glycosylation, the HRGP superfamily can be classified into three subfamilies: the hyperglycosylated arabinogalactan proteins (AGPs), the minimally glycosylated Pro-rich proteins (PRPs) and the moderately glycosylated extensins (EXTs) [5]. AGPs are abundant in plants, and can themselves be subdivided into six main subclasses: the classical AGPs, AG peptides, Lys-rich AGPs, FLAs, non-classical AGPs and chimeric AGPs [6]. FLAs generally have one or two fasciclin domains, and have been discovered in fruit flies, mammals, sea urchins, plants, yeast and bacteria. Besides fasciclin domains, FLAs often contain an N-terminal signal peptide as well as a C-terminal glycosylphosphatidylinositol (GPI) anchor signal peptide. The GPI and fasciclin domains are functionally important and are believed to mediate cell adhesion [7, 8].

So far, the FLA family members have been identified in several plant species. 21 *FLA*s have been identified in *Arabidopsis thaliana* [8], 27 in rice (*Oryza sativa*) [9, 10], 34 in wheat (*Triticum aestivum*) [10], 35 in poplar (*Populus trichocarpa*) [11], 19 in cotton (*Gossypium hirsutum*) [12], 33 in Chinese cabbage (*Brassica rapa*) [13], 18 in *Eucalyptus grandis* [14] and 23 in textile hemp (*Cannabis sativa*) [15]. FLAs are cell wall structural glycoproteins that mediate cellulose deposition and cell wall development. They are believed to participate in fiber development, elongation and stem dynamics, affecting the quality of fiber and wood in cotton and woody plants like poplar and eucalyptus [16] and are abundant in the xylem [17]. Knock down of *PtFLA6* resulted in a decrease of stem hardness and xylem cellulose lignin, and down-regulation of genes involved in cell wall synthesis [18]. Overexpression of *GhGalT1* promoted cotton fiber development by controlling the glycosylation of FLAs [19] and in plants where *GhAGP4* was knocked down, fiber initiation and elongation were strongly inhibited and there was suppression of the cytoskeleton network and of cellulose deposition in fiber cells [20]. During cell wall regeneration from cotton protoplasts, there is up regulation of proline-rich protein (PRPL), glycine-rich protein (GRP), and extensin (EPR1) but also of FLA2, which may mediate the construction and modification of the cell wall [21]. In addition, *AtFLA11*, *AtFLA12, EgrFLA2* and *EgrFLA3* have similar functions [14, 22]. *FLA*s can also regulate pollen development. In Arabidopsis and maize, *AtFLA9* and *ZmFLA7* showed negative correlation with abortion, and reductions in the expression of *FLA*s increased the abortion of fertilized ovaries [23]. *AtFLA3*-silenced Arabidopsis had abnormal pollen grains, also suggesting a function in pollen formation [24]. *FLA*s have also been implicated in cell-to-cell communication [13], shoot development [25, 26], seed mucilage adherence [27], glycan stabilization [28] and in response to stresses from salt [29–31], cold [32] and hydrogen peroxide [33].

Although *FLA*s have multiple roles in plant growth and development, very little is known about any involvement they may have in response to pathogens. *N. benthamiana* is a model plant for studying plant immunity, but the structure, function and expression of its *FLA* gene family members is unknown. In this study, we have identified and characterized the members of the *FLA* gene family in *N. benthamiana* and also reported their subcellular localization, expression patterns, and their response to viral and bacterial pathogens.

## Results

### Identification of members of the NbFLA family

Based on previous studies [8], FLAs have an AGP-like glycosylated region, a fasciclin domain and an N-terminal signal peptide. We followed these criteria to identify putative FLAs in *N. benthamiana*. The sequences of the 21 identified AtFLAs were downloaded [8] and the *N. benthamiana* genome was downloaded from the Sol Genomics Network (https://solgenomics.net/) [34]. A total of 38 NbFLAs were identified by two round BLASTP and signal peptide prediction (Table 1 and Additional file 1: Table S1). Most of these (66%) have lengths of 200-300aa, while the largest (NbFLA10) has 495aa and the smallest (NbFLA26) has only 182aa. The predicted isoelectric points range from 4.29 to 9.77, and the molecular weights (MWs) derived only from the amino acid sequences (not including glycans) are in the range 19.68–52.32 kDa. The protein properties of the NbFLAs are similar to those of other plant species [8, 11].

**Table 1:** Putative FLAs in *N. benthamiana*

gene symbol	gene locus	gene position	chromosome location	strand	CDS(bp)	protein length(aa)	Pi	amino acids MW(kDa)	predicted localization	FAS	GPI
NbFLA1	Niben101Scf00321g04012.1	Niben101Scf00321	Niben101Scf00321:422864,423997	+	1131	377	6.59	40.64	Cell membrane	2	N
NbFLA2	Niben101Scf00509g01008.1	Niben101Scf00509	Niben101Scf00509:85642,90123	-	741	247	6.39	26.08	Cell membrane	1	Y
NbFLA3	Niben101Scf00550g00014.1	Niben101Scf00550	Niben101Scf00550:46882,55345	+	1290	430	6.25	44.48	Cell membrane	1	N
NbFLA4	Niben101Scf00788g03029.1	Niben101Scf00788	Niben101Scf00788:460812,461639	+	825	275	4.62	28.95	Cell membrane, Nucleus	1	Y
NbFLA5	Niben101Scf00788g03035.1	Niben101Scf00788	Niben101Scf00788:422620,423441	-	819	273	4.74	28.55	Cell membrane	1	Y
NbFLA6	Niben101Scf00861g07009.1	Niben101Scf00861	Niben101Scf00861:810031,815156	+	1218	406	6.40	44.96	Cell membrane	2	N
NbFLA7	Niben101Scf00905g07001.1	Niben101Scf00905	Niben101Scf00905:720138,720957	-	774	258	4.80	26.97	Cell membrane	1	Y
NbFLA8	Niben101Scf01037g09001.1	Niben101Scf01037	Niben101Scf01037:932298,933101	+	801	267	5.60	28.17	Cell membrane	1	Y
NbFLA9	Niben101Scf01484g00009.1	Niben101Scf01484	Niben101Scf01484:43042,48168	+	1377	459	5.75	50.65	Cell membrane	2	N
NbFLA10	Niben101Scf01535g01003.1	Niben101Scf01535	Niben101Scf01535:172831,175164	-	1485	495	5.74	52.32	Cell membrane	2	Y
NbFLA11	Niben101Scf01685g06002.1	Niben101Scf01685	Niben101Scf01685:654403,655458	+	1053	351	7.76	38.87	Cell membrane	2	N
NbFLA12	Niben101Scf01911g00001.1	Niben101Scf01911	Niben101Scf01911:36923,37669	-	744	248	5.81	26.08	Cell membrane	1	Y
NbFLA13	Niben101Scf02994g01012.1	Niben101Scf02994	Niben101Scf02994:128443,129519	-	1074	358	4.40	39.73	Cell membrane	1	N
NbFLA14	Niben101Scf03271g02005.1	Niben101Scf03271	Niben101Scf03271:311394,312206	+	810	270	6.42	28.36	Cell membrane	1	Y
NbFLA15	Niben101Scf03563g00015.1	Niben101Scf03563	Niben101Scf03563:164934,165737	+	801	267	5.60	28.20	Cell membrane	1	Y
NbFLA16	Niben101Scf03634g11004.1	Niben101Scf03634	Niben101Scf03634:1153543,1157457	-	1344	448	6.14	49.40	Cell membrane	2	N
NbFLA17	Niben101Scf03757g02001.1	Niben101Scf03757	Niben101Scf03757:197902,202071	+	1377	459	5.76	50.79	Cell membrane	2	N
NbFLA18	Niben101Scf04444g06007.1	Niben101Scf04444	Niben101Scf04444:678486,679736	-	1248	416	5.93	43.84	Cell membrane	2	Y
NbFLA19	Niben101Scf04650g07004.1	Niben101Scf04650	Niben101Scf04650:711081,712304	+	1221	407	5.90	43.86	Cell membrane	2	Y
NbFLA20	Niben101Scf04731g11005.1	Niben101Scf04731	Niben101Scf04731:1262184,1263224	+	1038	346	9.41	37.88	Cell membrane	1	N
NbFLA21	Niben101Scf04765g02001.1	Niben101Scf04765	Niben101Scf04765:276562,277732	+	1080	360	5.44	38.75	Cell membrane	1	N
NbFLA22	Niben101Scf04792g02004.1	Niben101Scf04792	Niben101Scf04792:334985,335740	-	753	251	6.83	27.01	Cell membrane	1	Y
NbFLA23	Niben101Scf04813g00014.1	Niben101Scf04813	Niben101Scf04813:66883,69262	+	1386	462	5.50	48.34	Cell membrane	2	N
NbFLA24	Niben101Scf04847g00009.1	Niben101Scf04847	Niben101Scf04847:65900,67150	-	1248	416	5.81	44.05	Cell membrane	2	Y
NbFLA25	Niben101Scf05372g02008.1	Niben101Scf05372	Niben101Scf05372:251290,251817	+	750	250	6.03	26.10	Cell membrane	1	Y
NbFLA26	Niben101Scf05486g01006.1	Niben101Scf05486	Niben101Scf05486:176110,176890	+	546	182	9.23	19.67	Cell membrane	1	Y
NbFLA27	Niben101Scf05486g01015.1	Niben101Scf05486	Niben101Scf05486:168069,168845	+	774	258	6.06	27.36	Cell membrane	1	Y
NbFLA28	Niben101Scf06087g12013.1	Niben101Scf06087	Niben101Scf06087:1272274,1275168	+	1146	382	9.77	41.13	Cell membrane	1	N
NbFLA29	Niben101Scf06123g01025.1	Niben101Scf06123	Niben101Scf06123:103339,104049	+	708	236	8.79	25.61	Cell membrane	1	Y
NbFLA30	Niben101Scf06193g02005.1	Niben101Scf06193	Niben101Scf06193:268833,269871	+	747	249	9.36	27.50	Cell membrane	1	N
NbFLA31	Niben101Scf06203g03009.1	Niben101Scf06203	Niben101Scf06203:290365,293147	-	1326	442	6.26	47.82	Cell membrane	2	N
NbFLA32	Niben101Scf08300g00008.1	Niben101Scf08300	Niben101Scf08300:60937,61692	+	753	251	8.59	26.95	Cell membrane	1	Y
NbFLA33	Niben101Scf11969g00007.1	Niben101Scf11969	Niben101Scf11969:32722,34415	+	741	247	6.39	26.13	Cell membrane	1	Y
NbFLA34	Niben101Scf12431g00010.1	Niben101Scf12431	Niben101Scf12431:1378,2604	+	1224	408	5.41	43.98	Cell membrane	2	Y
NbFLA35	Niben101Scf13776g02002.1	Niben101Scf13776	Niben101Scf13776:333230,335138	+	1068	356	4.29	39.38	Cell membrane	1	N
NbFLA36	Niben101Scf19479g00006.1	Niben101Scf19479	Niben101Scf19479:91773,104152	-	819	273	9.63	29.11	Cell membrane	1	N
NbFLA37	Niben101Scf22195g00016.1	Niben101Scf22195	Niben101Scf22195:62492,63565	+	1071	357	8.56	39.46	Cell membrane	1	N
NbFLA38	Niben101Scf32632g00003.1	Niben101Scf32632	Niben101Scf32632:7941,10147	+	1128	376	5.30	41.56	Cell membrane	1	N

Notes: +: The mRNA sequence is positive strand; -: The mRNA sequence is negative strand; Y: The sequence has GPI signal; N: The sequence has no GPI signal.

### Phylogenetic analysis and multiple sequence alignment of NbFLAs

**Fig. 1:**
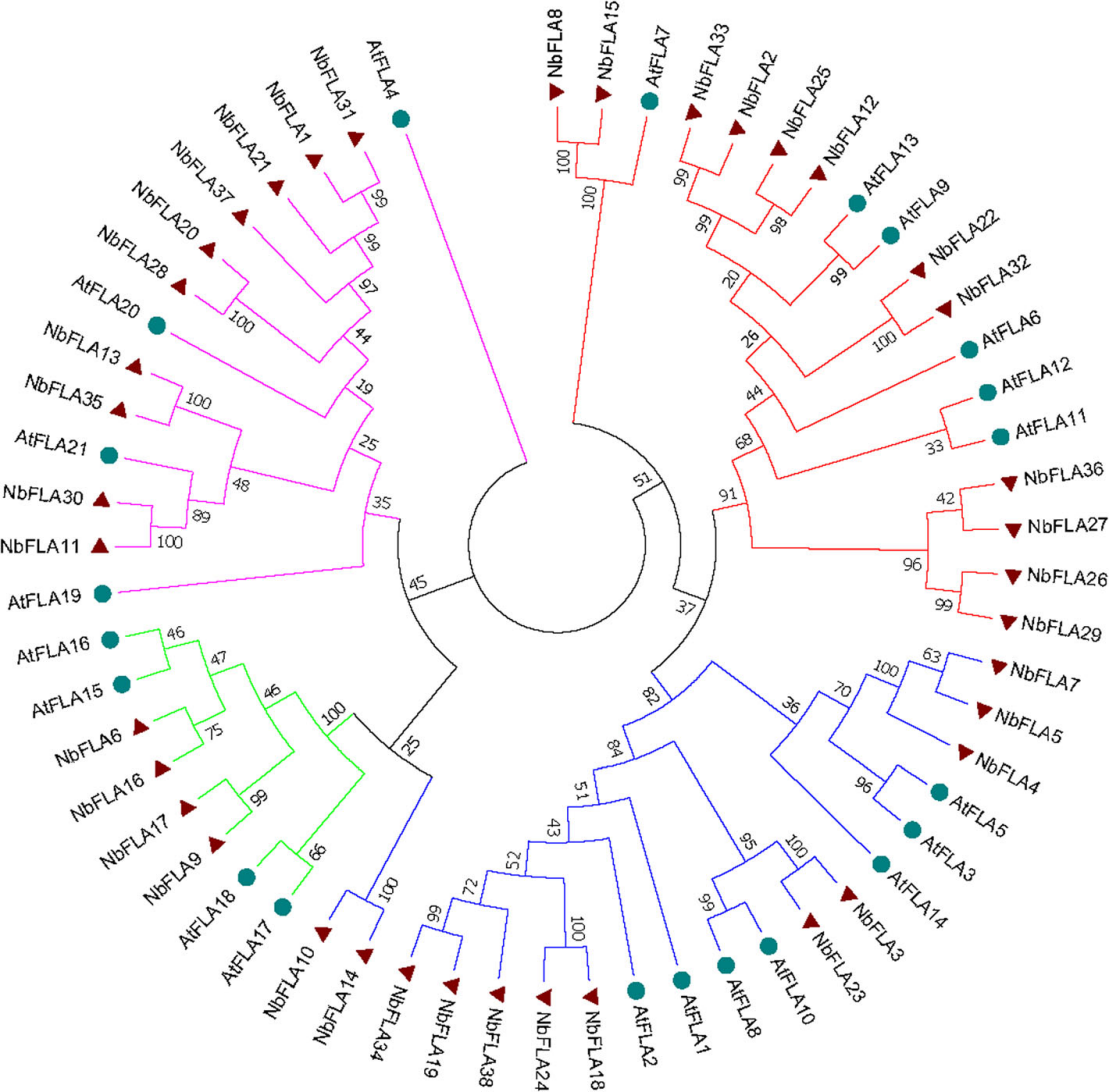
Unrooted phylogenetic tree representing relationships among FLA proteins of *N. benthamiana* and *A. thaliana*. All FLA proteins were divided into four subclasses represented by different colored clusters. Red, green, blue and pink clusters represent subclasses I, II, III and IV, respectively. The phylogenetic tree was constructed by the neighbor-joining method using MEGA7 software with 1000 bootstrap replicates

To better reveal their evolutionary relationships and to help the classification of NbFLAs, the sequences of all 21 AtFLAs and 38 NbFLAs were used to construct a phylogenetic tree (Fig. 1). Because of the low sequence similarity between some FLAs, phylogenetic analysis alone could be misleading and therefore pair-wise sequence similarity, presence and number of fasciclin domains and GPI were also used to create a classification, as previously described [8]. Most NbFLAs were sufficiently classified by phylogenetic analysis, but for a few (NbFLA8/15 and NbFLA10/14) their protein properties including the presence and number of fasciclin domains and GPI had also to be taken into account.

The 38 NbFLAs we identified could be divided into the same four subclasses previously reported for the AtFLAs [8], named I to IV (Fig. 1). NbFLA2/8/12/15/22/25/26/27/29/32/33/36 belong to subclass I, and have a single fasciclin domain and GPI anchored signal (except NbFLA36), as do the related AtFLAs and PtrFLAs [8, 11]. NbFLA6/9/16/17 belong to subclass II. Subclass II is the smallest group and members contain two fasciclin domains but have no C-terminal GPI anchor site. Members of subclass III (NbFLA3/4/5/7/10/14/18/19/23/24/34/38) have either one or two fasciclin domains, and most (77%) have a C-terminal GPI anchor site. The remaining NbFLAs (NbFLA1/11/13/20/21/28/30/31/35/37) constitute subclass IV, which contains NbFLAs that are quite distantly related to the other NbFLAs and which have no consistent pattern in the number of fasciclin domains or the presence of a GPI signal.

We also constructed separate phylogenetic trees for each subclass of NbFLAs, including the sequences from the other 8 plant species in which FLAs have been identified (Arabidopsis, rice, wheat, poplar, cotton, Chinese cabbage, *Eucalyptus grandis* and textile hemp) (Additional file 2: Fig. S1). In general, FLAs have a relatively high homology among closely related species, like AtFLAs/BrFLAs and OsFLAs/TaFLAs. FLAs from the same species often exist in pairs, like NbFLA26/29 and TaFLA19/27, suggesting that they may be paralogous genes. Subclasses I and III are the two largest groups and the clustering patterns are complicated. FLAs from the same species do not generally group together, and there are some closely-related pairs from different species suggesting that they are orthologous genes (e.g. NbFLA12/BrFLA22 and TaFLA2/OsFLA2). In subclasses II and IV, most FLAs from the same species group together (e.g. NbFLA6/9/16/17 and TaFLA6/7/8/29). Subclass II has fewest members and most of them are not GPI anchored, but the OsFLAs are a significant exception.

**Fig. 2:**
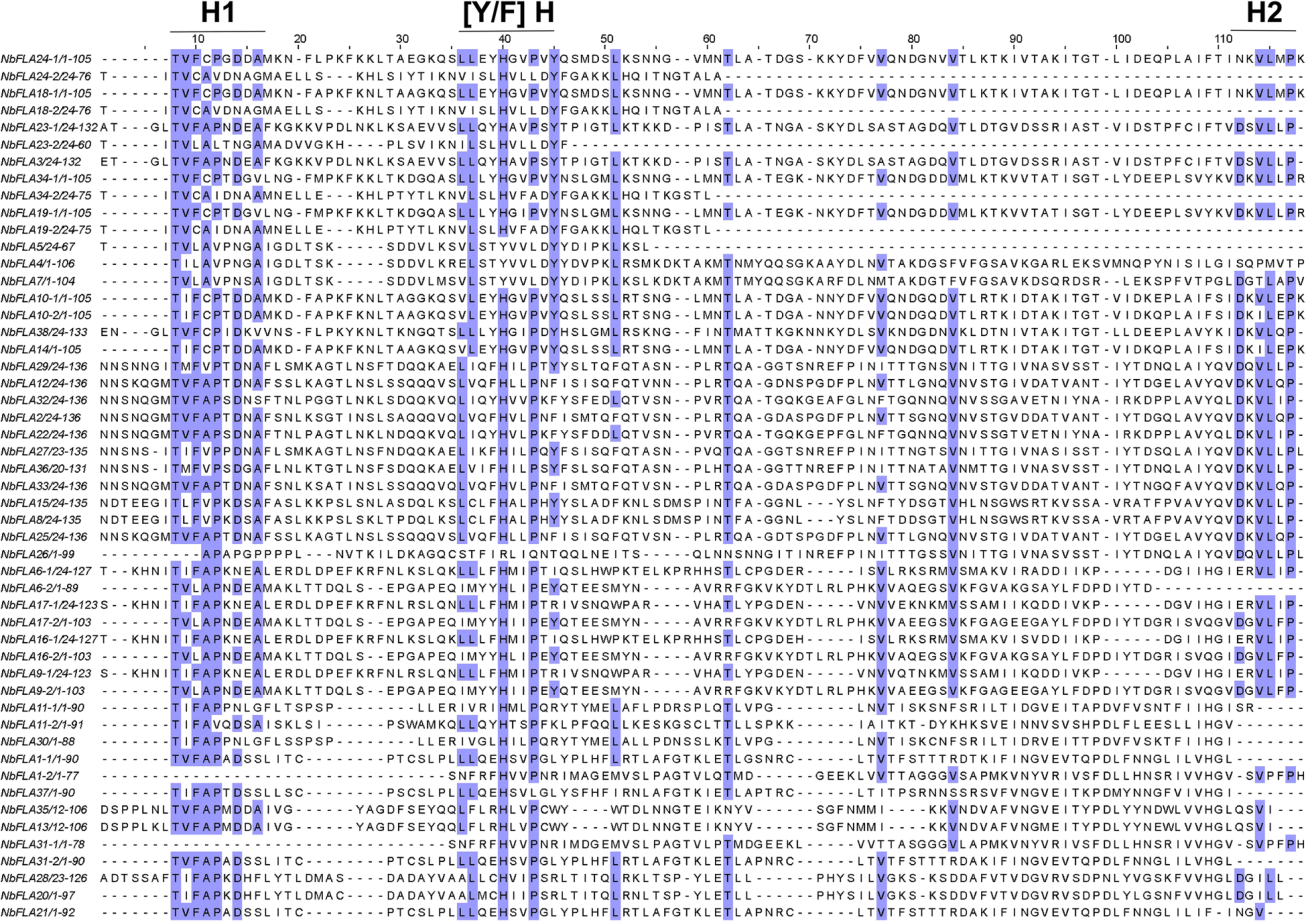
Multiple sequence alignment of the fasciclin domains of NbFLAs. The alignment was constructed by MUSCLE and visualized by Jalview. If an NbFLA contains two fasciclin domains, “-1” and “-2” are used to distinguish them. Residues in positions conserved more than 50% are shaded. Conserved regions (H1, H2, and [YF]H) are indicated at the top

Previously reported fasciclin domains contain about 110–150 amino acid residues and have two highly conserved regions (H1 and H2) and a [Phe/Tyr]-His ([Y/F] H) motif [12]. An alignment of the amino acid sequences of the fasciclin domains of the NbFLAs constructed using MUSCLE and some manual analysis showed a similar pattern (Fig. 2). The Thr residue in the H1 region is highly conserved and is followed by other conserved residues such as Val/ Ile (one position after Thr) and Asn/Asp (six positions after Thr). These residues may play a role in maintaining the structure of the fasciclin domain and/or cell adhesion [12]. As reported for other fasciclin domains [11, 31, 35], small hydrophobic amino acids such as Leu, Val and Ile are abundant in the H2 region. In the [Y/F] H motif, His and Pro residues are also relatively conserved.

### Analysis of the structural and conserved motifs of *NbFLAs*

**Fig. 3:**
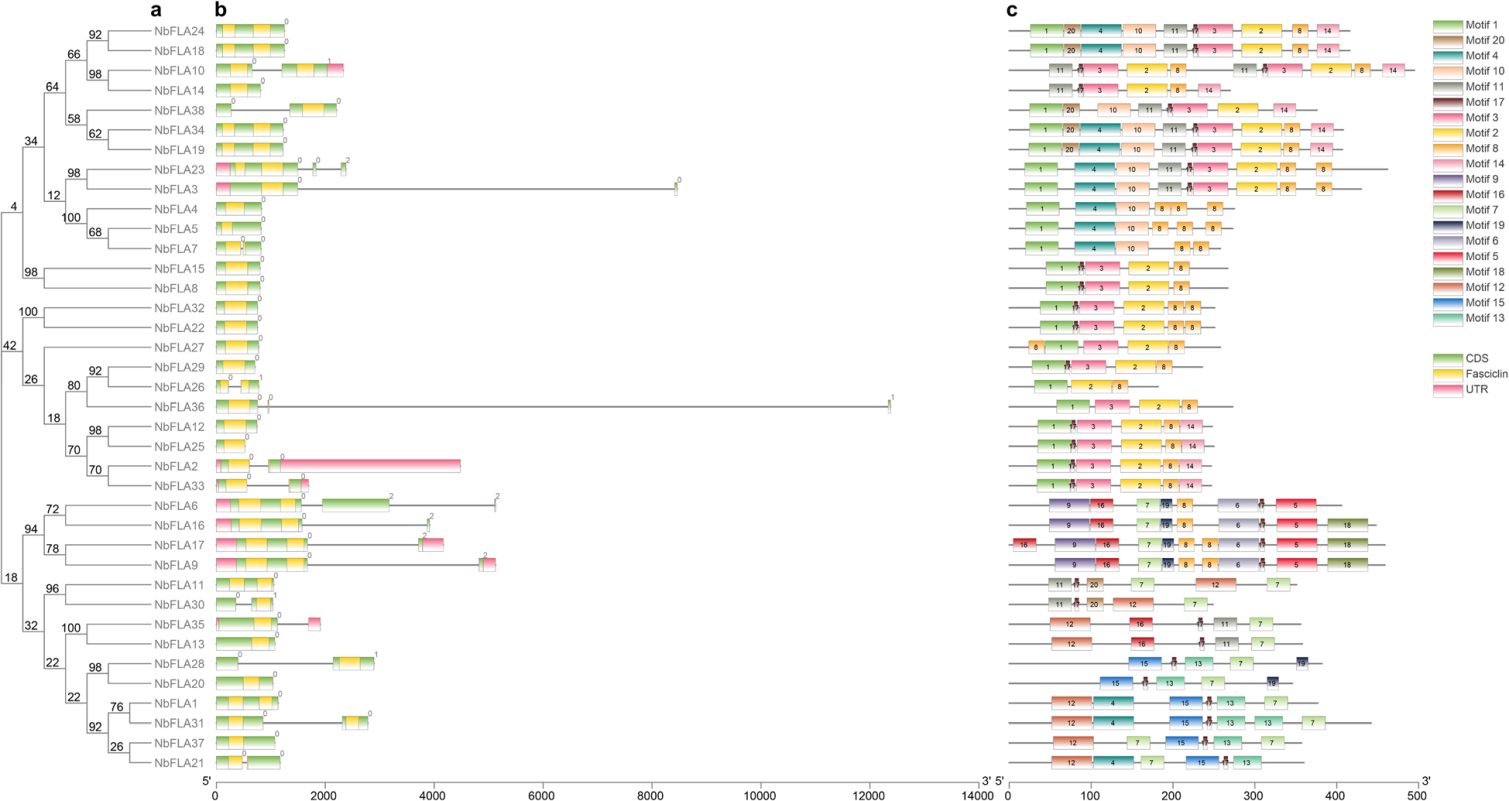
Phylogenetic relationship, gene structure and architecture of the conserved protein motifs in NbFLAs. **a** The phylogenetic tree was constructed based on the full-length sequences of NbFLA proteins. **b** Exon-intron structure of *NbFLAs*. Pink boxes indicate untranslated 5′- and 3′-regions; green boxes indicate exons; and black lines indicate introns. The fasciclin domains are shown by yellow boxes. **c** The motif composition. The motifs, numbered 1–20, are displayed in different colored boxes. The sequence information for each motif is provided in Additional file 1: Table S2

Further analysis of gene structure and motifs of the NbFLAs is shown in Fig. 3. The phylogenetic tree confirmed that NbFLAs could be grouped into four subclasses (Fig. 3a). Analysis of the genomic DNA sequences showed that *NbFLAs* usually had 0, 1 or 2 introns (Fig. 3b). All of the members in subclass II have one or two introns while most members of subclasses I and III have none (Fig. 3b). The most closely related members of each subclass, usually have a similar exon/intron structure, with little difference in the length of introns and exons. However, a few *NbFLA* gene pairs showed different intron/exon arrangements. For example, *NbFLA1* and *NbFLA31* have high sequence similarity, but *NbFLA1* has no introns while *NbFLA31* has one.

An online MEME analysis was done to identify additional motifs among the 38 NbFLAs. Twenty conserved motifs were predicted (Fig. 3c and Additional file 3: Table S2) and each NbFLA contained between five and ten of these. Some motifs were common to most members, while the others were unique to one or few subclasses. For example, most NbFLAs (84%) contained motif 17. Motifs 10 and 11 were present only in subclass III and motifs 9, 16, 18 and 19 were found only in subclass II. Motif 7 was unique to subclasses II and IV, and most members of subclasses I and III contained both motifs 3 and 8 except NbFLA4/5/7/26/38. Subclass IV was clearly less closely related to the other subclasses, and motifs 12, 13 and 15 were unique to this subclass.

### Prediction of cis-acting elements and transcription factors among the *NbFLAs*

**Fig. 4:**
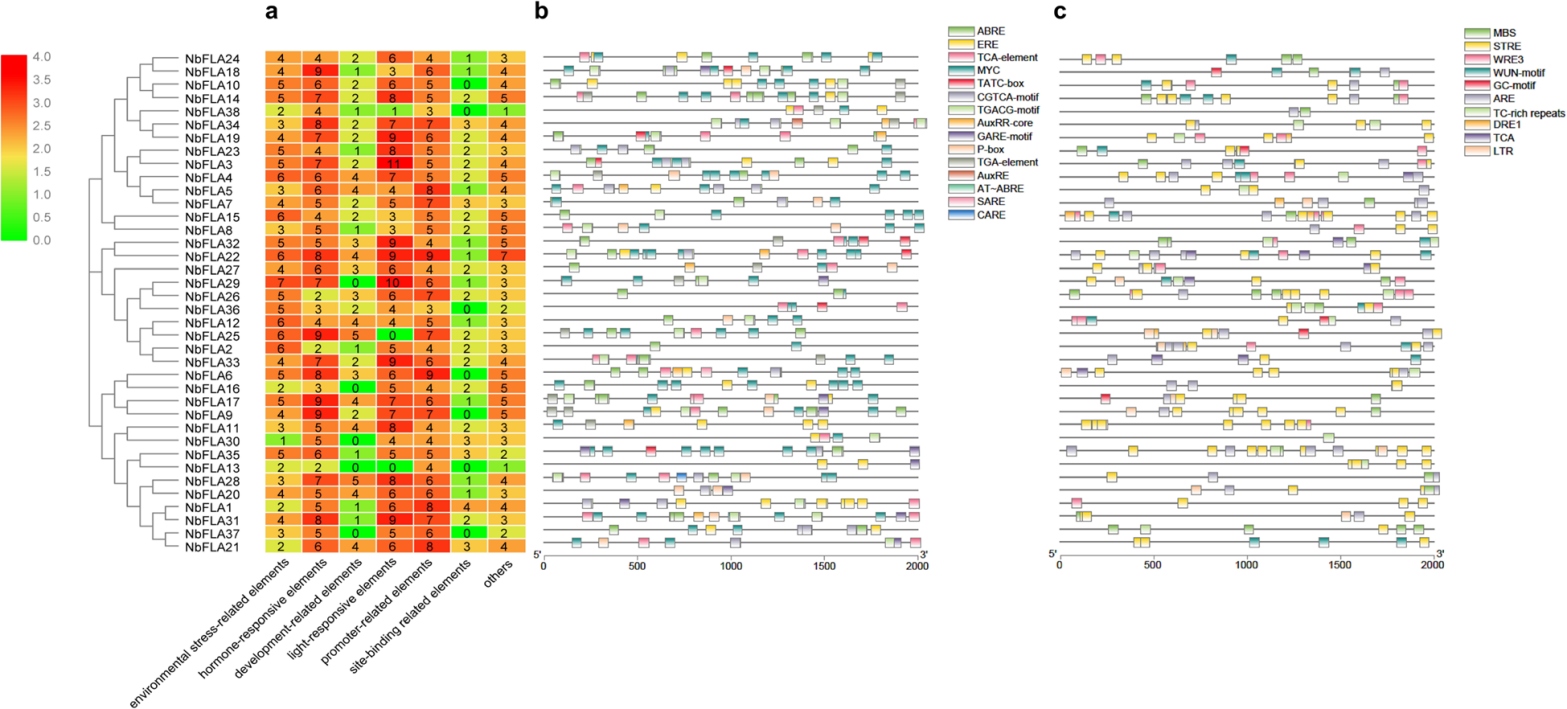
Prediction of cis-acting elements in *NbFLAs*. **a** numbers of cis-acting elements detected in the promoter region of each *NbFLA* gene. All cis-acting elements were divided into seven types. **b** Kind, quantity and position of environmental stress-related elements in *NbFLAs*. **c** Kind, quantity and position of hormone responsive elements in *NbFLAs*

The cis-acting elements in the promoter regions of the NbFLAs were analyzed and a totally 105 cis-acting elements were predicted (Fig. 4 and Additional file 4: Table S3). These cis-acting elements were related to environmental stress, hormone response, development, light response, promoter, site binding and other functions (Fig. 4a). The most abundant elements were light-responsive elements, including G-box, GT1-motif and GATA-motif. 15 hormone responsive elements were identified and these are mainly involved in response to abscisic acid (ABA) or methyl jasmonate (MeJA) (Fig. 4b). Among the predicted environmental stress-related elements, STRE, MBS and ARE were the most abundant (Fig. 4c). Several abundant predicted cis-acting elements are known to mediate plant immunity. For example, *VdMYB1* binds to the MBS in the *VdSTS2* gene promoter, thus activating *VdSTS2* transcription and positively regulating defense responses [36]. *Machi3–1* and *TaRIM1* also bind MBS cis-acting elements to increase host resistance [37, 38].

**Fig. 5:**
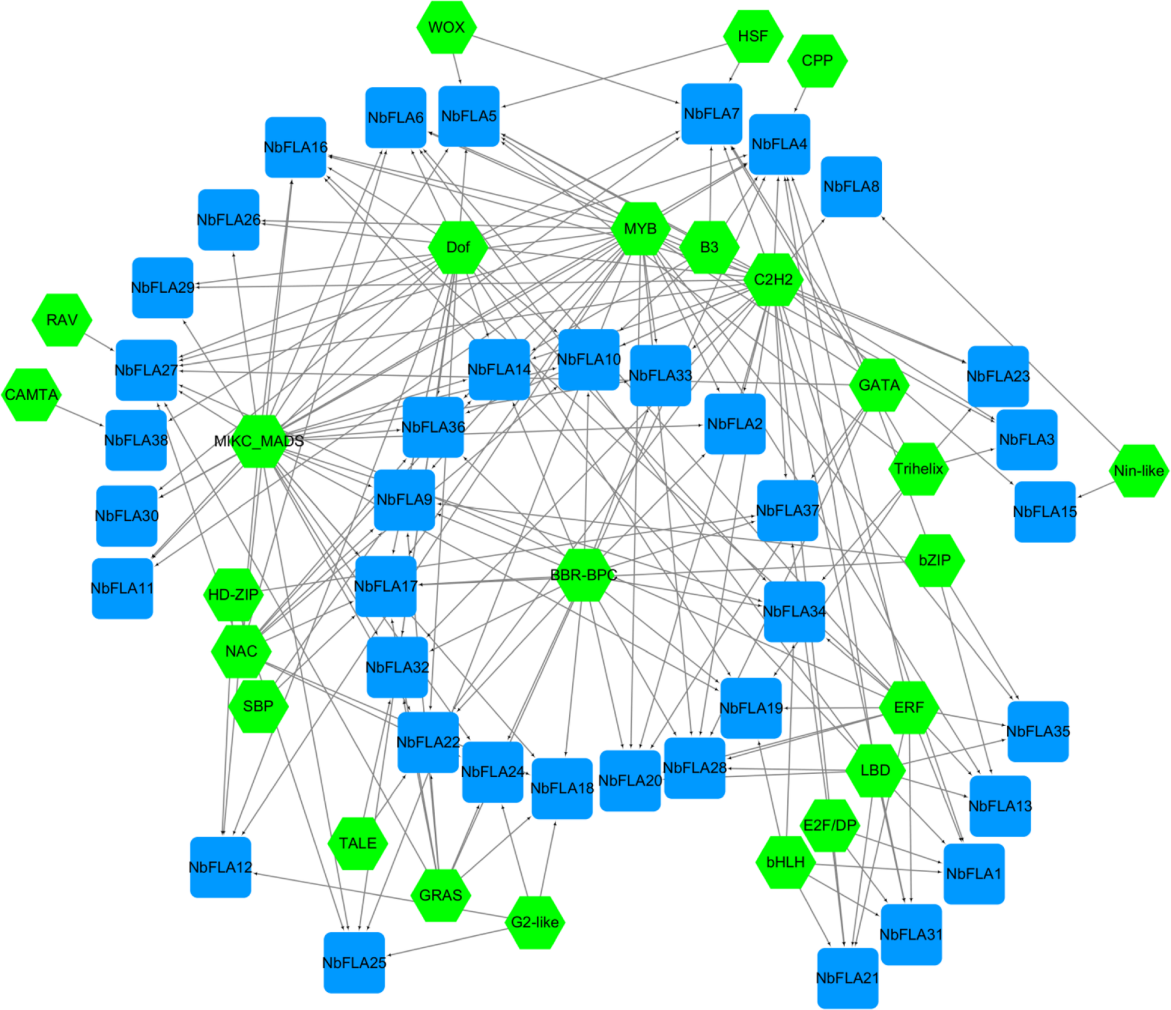
Regulation network between *NbFLAs* and potential TFs. Green hexagons represent transcription factors, blue rectangles represent *NbFLAs*, and black lines represent potential regulatory relationships

By binding to transcription factors (TFs), cis-acting elements regulate the precise initiation and efficiency of gene transcription. We then therefore predicted potential TFs which may regulate the transcription of *NbFLAs* (Fig. 5 and Additional file 5: Table S4). The *NbFLAs* had an average of five TFs, but it appears that *NbFLA4* and *NbFLA27* may be regulated by more TFs, including specific TFs like RAV and CPP, while *NbFLA8/15/38* may each be regulated by only two TFs. In total, 25 TFs were predicted of which C2H2, BBR-BPC, Dof, Myb and MIKC were the most abundant. Previous studies have demonstrated the role of TFs in regulating plant immunity. NbCZF1, a novel C2H2-Type zinc finger protein, is a regulator of plant defense [39] and VvDOF3 enhances powdery mildew resistance in *Vitis vinifera* [40]. In addition, AtMyb15 and MdMyb30 also participate in enhancing disease resistance [41, 42].

### Subcellular localization analysis of NbFLAs

**Fig. 6:**
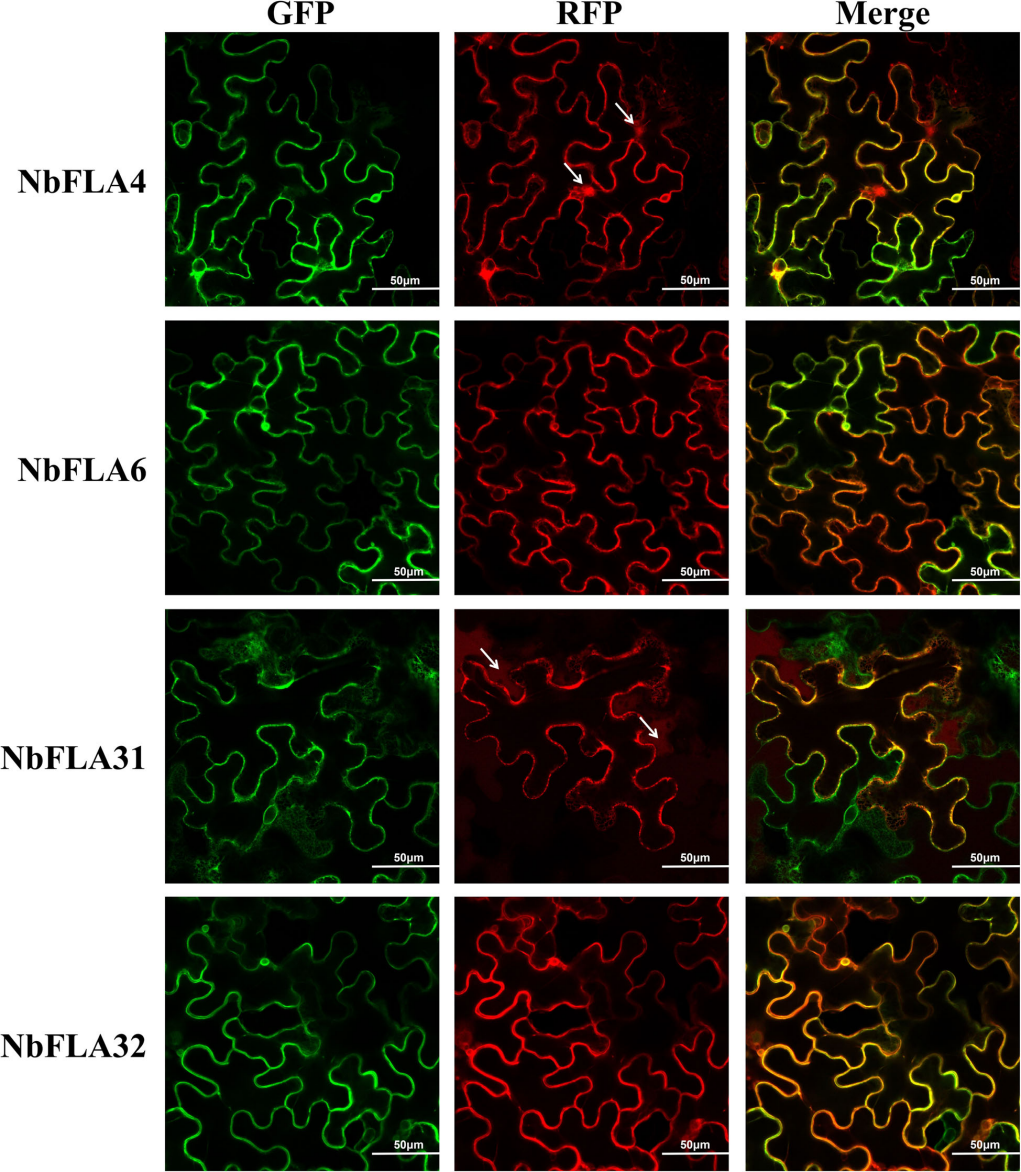
Subcellular localization of NbFLA4/6/31/32. Confocal microscopy images of *N. benthamiana* epidermal leaf cells co-expressing the membrane marker AtP1P2A-GFP (left panels) with NbFLA4-mCherry, NbFLA6-mCherry, NbFLA31-mCherry and NbFLA32-mCherry (middle panels), respectively. Merged images are shown in the right panels. Scale bars = 50 μm. Arrows in the panel of NbFLA4-mCherry indicate red fluorescence in the nucleus. Arrows in the panel of NbFLA31-mCherry indicate red fluorescence in the cytoplasm

Bioinformatics analysis based on the NbFLA amino acid sequences suggested that all of them could locate to membranes, and only NbFLA4 was predicted to locate in both the nucleus and membranes (Table 1). To validate these predictions, we selected one NbFLA in each subclass (NbFLA4/6/31/32) to analyze their localization by laser confocal microscopy. AtP1P2A-GFP was used as membrane marker [43]. The results showed that while NbFLA6 and NbFLA32 were only located in membranes, NbFLA4 was present both in membranes and the nucleus, consistent with the predictions (Fig. 6).

A GPI anchored signal is vital for membrane localization and is predicted in about two thirds of AtFLAs and PtrFLAs and in 20 of 38 (53%) of NbFLAs (Table 1). Among the four selected NbFLAs, only NbFLA31 was not GPI anchored. Correspondingly, although a plasmolysis experiment confirmed the membrane localization of NbFLA31, a diffused red fluorescence could also be observed in the cytoplasm (Fig. 6 and Additional file 6: Fig. S2).

### Tissue-specific expression of *NbFLAs*

**Fig. 7:**
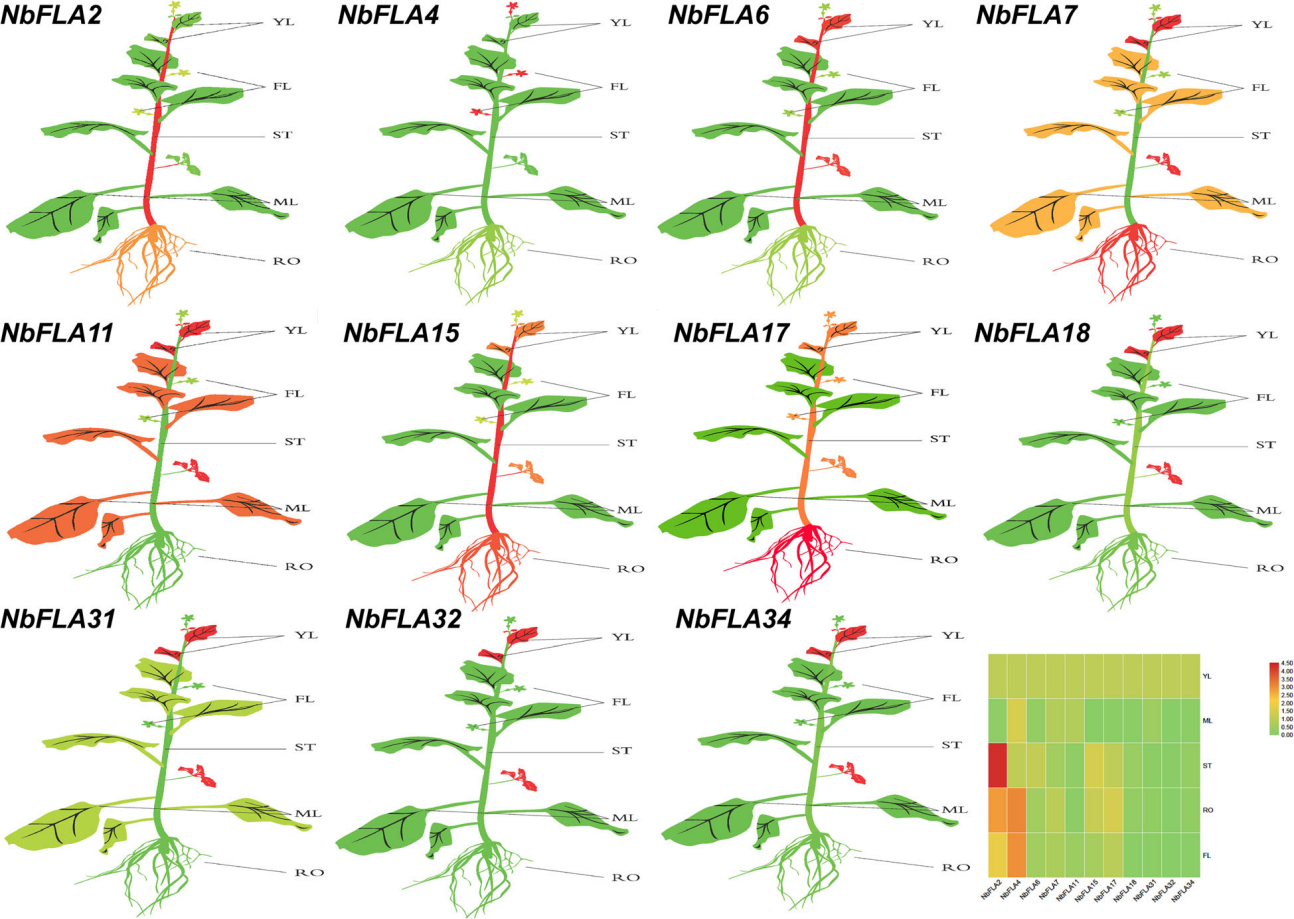
The differential expression of representative *NbFLA* genes in different tissues by RT-qPCR. YL: young leaf; MF: mature leaf; ST stem: RO root; FL: flower. The mean expression value was calculated from three independent biological replicates relative to that in young leaves. The mean expression values were visualized by Tbtools; red represents high expression level and green represents low expression level. The raw data of relative expression values and standard errors is provided in Additional file 6: Fig. S2

To comprehensively understand the functions of *NbFLAs*, two or three *NbFLAs* from each subclass were randomly selected to analyze their expression in five different tissues (root, stem, young leaf, mature leaf and flower) by RT-qPCR (Fig. 7 and Additional file 7: Fig. S3). The expression level of all selected *NbFLAs* (except *NbFLA4*) was higher in young leaves than in mature ones. *NbFLA11/18/31/32/34* were highly expressed in young leaves, and *NbFLA4* were expressed highly in flowers. It was earlier reported that *PtFLA6* is specifically expressed in tension wood (TW) and that decreased transcripts of *PtFLA6* influenced stem dynamics [18]. In this study, *NbFLA2/6/15/17*, belonging to subclasses I and II, were highly expressed in stems, suggesting that they may play a role in stem dynamics.

### Expression of *NbFLA*s under biotic stress

**Fig. 8:**
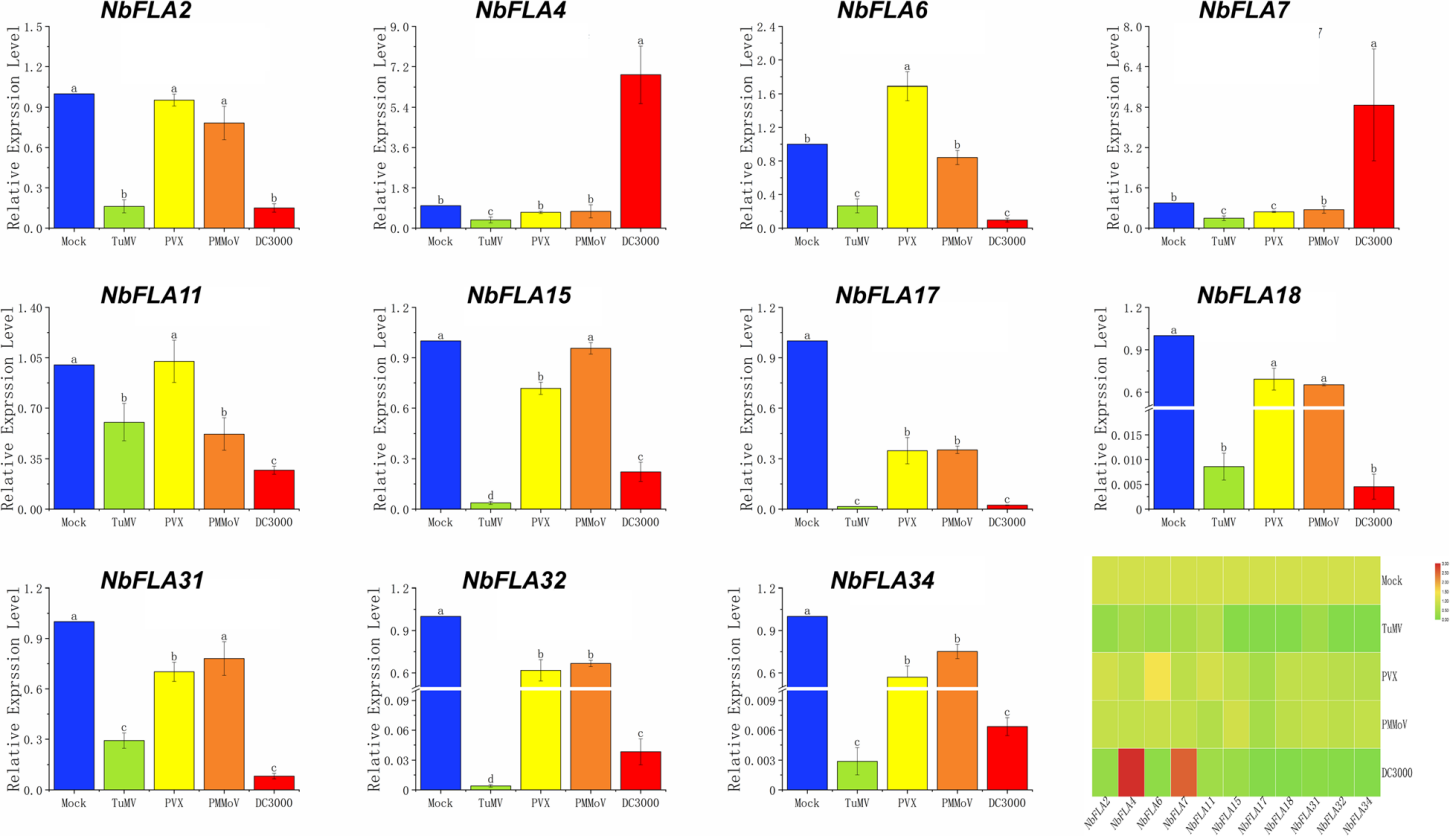
Expression analysis of representative *NbFLA* genes infected with different pathogens by RT-qPCR. The mean expression values were calculated from three independent biological replicates and are relative to mock-inoculated controls

To investigate whether *NbFLAs* participate in the response to pathogens, leaves of *N. benthamiana* were inoculated with turnip mosaic virus (TuMV), potato virus X (PVX), pepper mottle mosaic virus (PMMoV) and the bacterial pathogen *Pseudomonas syringae pv tomato* strain DC3000 (*Pst* DC3000). At 5 days post virus inoculation (dpi), or 2 days post *Pst* DC3000 infection, leaves were collected to study the expression pattern of 11 *NbFLA* genes by RT-qPCR (Fig. 8).

TuMV infection led to a huge reduction in expression of all the *NbFLAs* tested, especially *NbFLA15/18/32/34,* which all decreased by more than 99%. PVX or PMMoV infection usually induced a modest reduction in expression, although *NbFLA6* was slightly upregulated by PVX. The bacterial pathogen *Pst* DC3000 decreased expression of most *NbFLAs* by 73–99% but, in contrast, *NbFLA4* and *NbFLA7* were substantially upregulated. These results show that most *NbFLAs* are substantially affected by TuMV and *Pst* DC3000 and may therefore play roles in post-infection responses.

## Discussion

*FLA* families have been identified and characterized in several plants including Arabidopsis [8], rice [9, 10], wheat [10], poplar [11], cotton [12], Chinese cabbage [13], *Eucalyptus grandis* [14] and textile hemp [15]. In this study, we identified 38 *FLA*s in *N. benthamiana* and found that their structural domains were conserved by studying phylogenetic trees, gene structure and conserved motifs (Fig. 3). In general, NbFLAs could be divided into four subclasses and NbFLAs in each subclass had similar gene structure, motifs and conserved domains. Consistent with the FLAs in Arabidopsis [8], subclass II contained fewest NbFLAs and NbFLAs in subclass IV were the most variable. The FLAs of other dicotyledonous plant species had similar properties in each subclass, but while dicot members of subclass II have no GPI, most OsFLAs and TaFLAs in the subclass are GPI anchored [10]. In addition, OsFLAs in subclass II have only one fasciclin domain, unlike the FLAs of the dicotyledonous species [10]. Thus a different classification of FLAs in monocotyledonous plants may be required.

Twenty-five of the 38 NbFLAs had a single fasciclin domain, 13 of them had two domains and 20 of the 38 were GPI anchored. A GPI-anchored signal together with a fasciclin domain are known to be important for cell adhesion, for membrane localization and for enabling more stable interactions between adhesion complexes. It has been suggested that plants may have FLAs with GPI-anchoring for maintaining the integrity of the plasma membrane and FLAs that are not GPI-anchored for mediating cell expansion [8].

Previous studies have shown different expression patterns of *FLAs* in the tissues of other plants. For example, *AtFLA11/12* were highly expressed in stems [22], as were *BrFLA6/9/22* (homologous to *AtFLA11*)*.* Some *EgrFLAs* were also highly expressed in stems [14, 22] and 10 *PopFLAs* were highly expressed in poplar tension wood [35]. *PtFLA6* and *ZeFLA11* were exclusively expressed in xylem tissues [18, 44]. These studies suggest that some *FLAs* play important roles in stem dynamics and cell wall elongation. In our study, *NbFLA2/6/15* were also expressed highly in stems whereas *NbFLA7/34* were highly expressed in roots, as were *PtrFLA12/21/22/24/27/28/30* [11], indicating that they may participate in root apical meristem development. Many *NbFLAs* were expressed highly in young leaves [11], as reported for *GhFLA5/8/9/12* and *Br4/5/10/21/27/33* [8, 12, 13], but no *PtrFLAs* tested had high expression in young leaves [11]. This may be because *N. benthamiana* more closely resembles cotton and Chinese cabbage in being a herbaceous annual.

Some biotic and abiotic stresses lead to significant changes in the transcription of *FLAs*. For example, Under H_2_O_2_ stress, the expression levels of wheat FLA proteins were increased, which may contribute to H_2_O_2_ tolerance [33]. Similarly, *AtFLA3* was expressed more highly under cold stress [32]. Under salt stress, *OsFLA10/18* expression was reduced [9] while *PtrFLA2/12/20/21/24/30* were upregulated [11]. In addition, T*aFLA3/4/9* were downregulated after heat, ABA or NaCl treatment [10]. *OsFLA24* and *AtFLA1/2/8* were also significantly reduced following ABA treatment [8, 9]. Many of the frequently predicted TFs in the *NbFLAs*, including C2H2, Dof and Myb, have been reported to play a role in the ABA pathway [45–48] and therefore, as in other species, *NbFLAs* may be regulated by the ABA pathway. While the function of *FLAs* in the signaling pathway during abiotic stresses has been investigated, little is known about their potential role in response to pathogens. *AtFLA1/2/8* were decreased by pathogen challenge, oxidative stress and in ascorbate-deficient vtc mutants [49]. The fungus *Ophiostoma novo-ulmi* reduced the expression of *FLAs* in English elm ramets [50]. Our results show that almost all *NbFLAs* were specifically downregulated by TuMV and *Pst* DC3000 infection and this suggests that *NbFLAs* may have specific roles in pathogen infection.

Because of their role in cell adhesion and their membrane localization, AGPs (including FLAs) may interact with receptor-like kinases as wall-associated kinases and thus be involved in signal transduction [51]. For example, *AtFLA4* (*SOS5*) mediated root growth and seed adhesion through cell wall receptor-like kinase (*FEI1/2*) [27], and modulated ABA signaling to regulate cell wall biosynthesis and root growth [25, 27]. The known functions of GPI and the fasciclin domain suggest that NbFLAs might be involved in host-pathogen interactions. Thus, a further role of *NbFLAs* in plant resistance is worth exploring.

## Conclusion

In this study, 38 *NbFLAs* were identified and could be divided into four subclasses. In general, the closest members of NbFLAs from the same subclass have similar structure and conserved motifs. The expression patterns of selected *NbFLA*s in different tissues were diverse and selected *NbFLAs* were downregulated following infection by TuMV or *Pst* DC3000. Our results will help to lay the foundation for understanding of the structure and characteristics of the FLA family and for exploring the relationship between *FLAs* and immunity in *N. benthamiana.*

## Methods

### Identification of the NbFLAs family

The sequences of the 21 identified AtFLAs were downloaded and the *N. benthamiana* genome was downloaded from the Sol Genomics Network (https://solgenomics.net/) [34]. NbFLAs were identified by two rounds of BLASTP. Firstly, all AtFLAs were used to search possible NbFLAs using TBtools [52]. Then NCBI Batch CD-Search [53, 54] was used to confirm whether candidate NbFLAs contained a fasciclin domain including FAS1 (smart00554), Fasciclin superfamily (cl02663) or Fasciclin (pfam02469). Next, we predicted the N-terminal signal peptide by SignaIP5.0 [55], the C-terminal GPI anchor addition signal by big-PI Plant Predictor [56], and the glycosylation site by NetGlycate 1.0 [57]. Finally, using criteria previously established, sequences that contained an AGP-like glycosylated region, fasciclin domains and an N-terminal signal peptide were considered as NbFLAs [11]. The CDS length, pI and molecular weights (MW) of all predicted NbFLAs were then determined by ExPASy [58] and their subcellular localization predicted by Plant-mPLoc [59].

### Phylogenetic analysis and multiple sequence alignment

Sequences of AtFLA proteins were obtained from the NCBI protein database (http://www.ncbi. nlm.nih.gov/protein/). A neighbor-joining (NJ) phylogenetic tree of full-length sequences of AtFLAs and NbFLAs was constructed with 1000 bootstrap replicates using MEGA7.0. A multiple sequence alignment of all NbFLAs was also created by Clustal X 2.0 [60].

### Gene structure and conserved domain analysis

Gene structure and conserved domains were analyzed and visualized using NCBI Batch CD-Search [53, 54] and TBtools [52]. Conserved motifs of the genes were analyzed by the MEME program [61] with the following parameters: optimum motif width was set to 30–70, the number of repetitions was set to zero or one, the maximum number of motifs was set to identify 15 motifs.

### Promoter cis-acting elements and TFs prediction

The promoter cis-Acting elements were predicted by PlantCARE [62] and transcription factors were predicted by PlantRegMap [63], with *N. sylvestris* as the target species.

### Plasmid construction and Agroinfection assays in *N. benthamiana*

Based on the sequences above, we cloned the CDS sequences of *NbFLA4/6/31/32* and constructed them into a transient expression vector with red fluorescent label. All primers used for plasmid construction are listed in Additional file 8: Table S5. Agroinfection assays were conducted as previously described [64]. Briefly, the constructs were transformed into *A. tumefaciens* (strain GV3101) by electroporation. The transformants were cultured and re-suspended in the inoculation buffer [10 mM MgCl_2_, 2 mM acetosyringone, 100 mM MES (pH 5.7)] for 3-5 h at room temperature. The suspensions were then adjusted to OD_600_ = 0.1 and were infiltrated into leaves of 4- to 6-week old *N. benthamiana* plants with needleless syringes.

### Plant growth and pathogen inoculation

*N. benthamiana* seeds were donated by Dr. Yule Liu (Tsinghua University, China) and grown in mixed soil matrix (peat: vermiculite = 1:1) under a 16-h light (2000 lx)/8-h dark photoperiod at 26 ± 2 °C with relative humidity 60 ± 5%. A TuMV infectious clone was kindly provided by Dr. Fernando Ponz (INIA, Laboratorio de Virologı’a Vegetal, Spain), a PVX infectious clone was kindly provided by Dr. Stuart MacFarlane (James Hutton Institute, UK) and a PMMoV infectious clone was created in our lab. The *Pst* DC3000 strain was kindly provided by Dr. Yule Liu (Tsinghua University, China). TuMV, PVX and PMMoV were inoculated onto the newly expanded leaves of *N. benthamiana*. Inoculum was obtained by homogenizing virus-infected leaves in phosphate buffer, and with phosphate buffer as mock control. The *Pst* DC3000 was cultured in King’s B medium at 28 °C. Leaves of *N. benthamiana* were infiltrated with a suspension of *Pst* DC3000 (OD_600_ = 10^− 5^) in 10 mM of MgCl_2_, while plants only infiltrated with 10 mM of MgCl_2_ were used as the negative control as previously described [65]..

### Expression analysis by RT-qPCR

RT-qPCR analysis was performed to confirm the expression of representative *NbFLA* genes. We used at least three independent biological replicates and three technical replicates. First-strand cDNA was synthesized from 0.5 mg of RNA with PrimeScript RT reagent kit (TaKaRa). RT-qPCR was carried out by SYBR-green fluorescence using the Roche LightCycler®480 Real-Time PCR System. Relative gene expression levels were calculated according to the ΔΔCT method [66] and visualized in a heat map by Tbtools [52]. All primers used for RT-qPCR are listed in Additional file 8: Table S5.

## Supplementary information

**Additional file 1: Table S1.** List of NbFLA CDS and protein sequences.

**Additional file 2: Figure S1.** Unrooted phylogenetic trees showing the relationships among FLA proteins of 9 plant species in each subclass. a, b, c, d represent subclasses I, II, III, IV, respectively. The phylogenetic trees were constructed by Neighbor-joining using MEGA7 software and with 1000 bootstrap replicates.

**Additional file 3: Table S2.** The MEME motif sequences and length of NbFLAs.

**Additional file 4: Table S3.** Cis-acting elements in *NbFLAs*.

**Additional file 5: Table S4.** Potential transcription factors of *NbFLAs*.

**Additional file 6: Figure S2.** Plasmolysis experiment of NbFLA31. Confocal microscopy images of *N. benthamiana* epidermal leaf cells expressing NbFLA31-mCherry. Plasmolysis was induced using a 20% NaCl hypertonic solution. Arrows indicate visual plasmolysis spaces. Scale bars = 50 μm.

**Additional file 7: Figure S3.** The differential expressions of representative *NbFLA* genes in different tissues by RT-qPCR (raw data). YL: young leaf; MF: mature leaf; ST: stem; RO: root; FL: flower. The mean expression values were calculated from three independent biological replicates and are relative to that in young leaves.

**Additional file 8: Table S5.** Primers used in this study.

## Data Availability

All data generated or analyzed during this study are included in this published article and its Additional files. The datasets generated and analyzed during the current study are available from the corresponding author on reasonable request.

## Abbreviations

FLAs: Fasciclin-like arabinogalactan proteins
AGPs: Arabinogalactan proteins
GPI: Glycosylphosphatidylinositol
TuMV: Turnip mosaic virus
*Pst* DC3000: *Pseudomonas syringae pv tomato* (*Pst*) strain DC3000
HRGPs: Hydroxyproline-rich glycoproteins
TFs: Transcription factors
ABA: Abscisic acid
MeJA: Methyl jasmonate
TW: Tension wood
PVX: Potato virus X
PMMoV: Pepper mottle mosaic virus
FAS1: Fasciclin 1
