# Supplementary figures and images for: Fasciclin-like arabinogalactan gene family in Nicotiana benthamiana: genome-wide identification, classification and expression in response to pathogens
Source: BMC Plant Biol. 2020 Jul 1;20:305. doi: 10.1186/s12870-020-02501-5 (PMC7329489; doi:10.1186/s12870-020-02501-5)

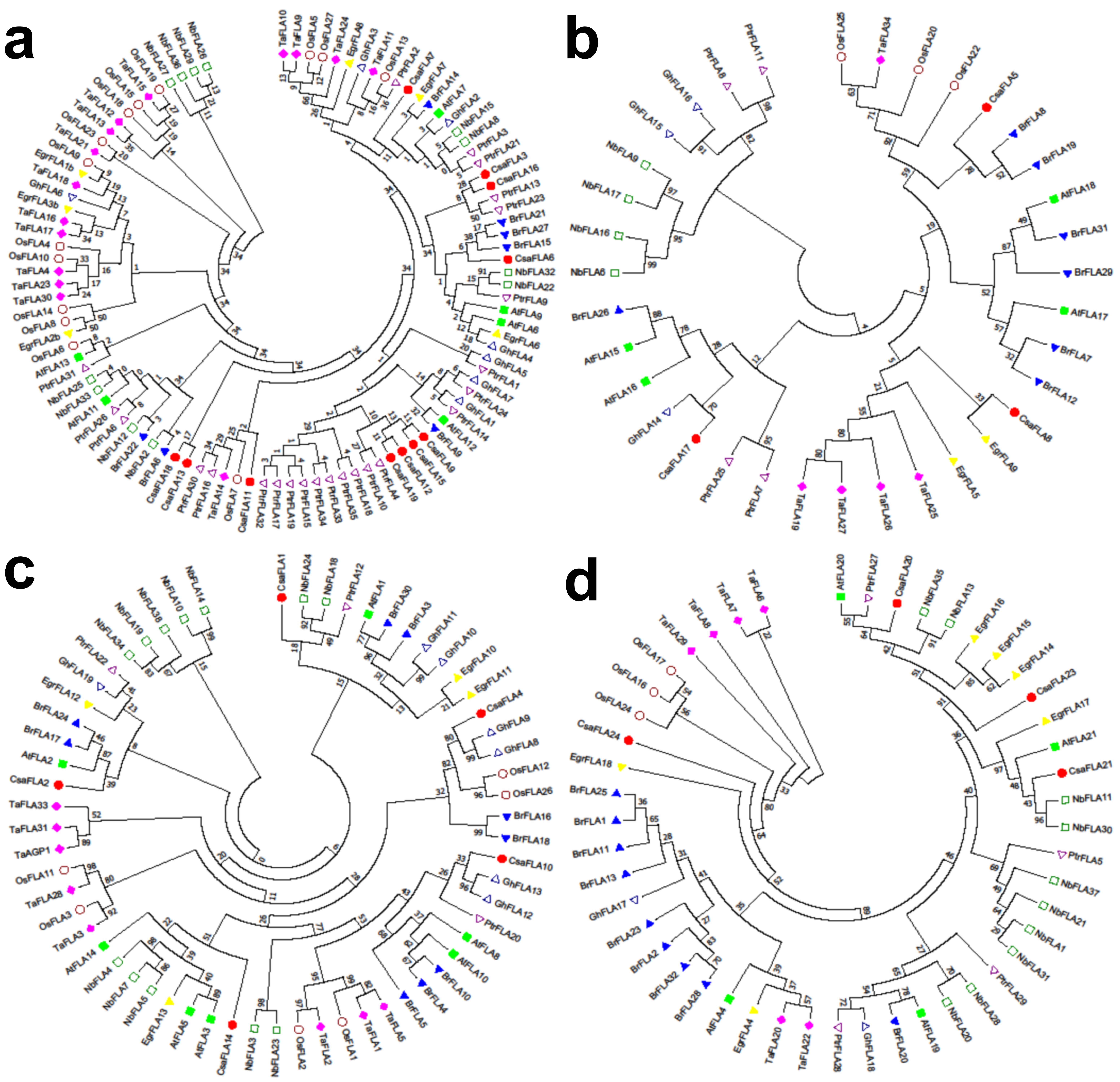

Supplement: Supplementary file 2 — Additional file 2: Figure S1. Unrooted phylogenetic trees showing the relationships among FLA proteins of 9 plant species in each subclass. a, b, c, d represent subclasses I, II, III, IV, respectively. The phylogenetic trees were constructed by Neighbor-joining using MEGA7 software and with 1000 bootstrap replicates. [file 12870_2020_2501_MOESM2_ESM.jpg]

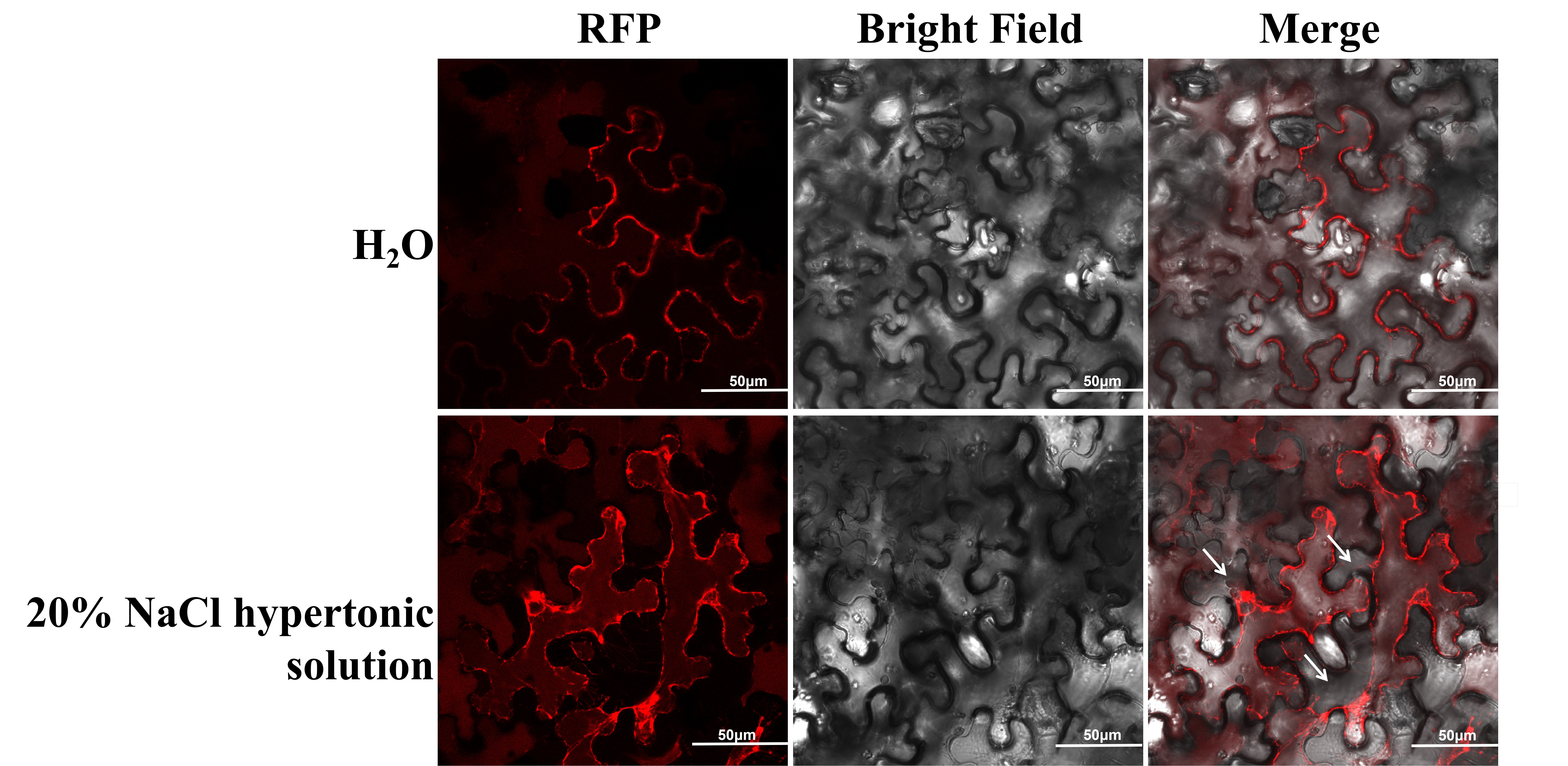

Supplement: Supplementary file 6 — Additional file 6: Figure S2. Plasmolysis experiment of NbFLA31. Confocal microscopy images of N. benthamiana epidermal leaf cells expressing NbFLA31-mCherry. Plasmolysis was induced using a 20% NaCl hypertonic solution. Arrows indicate visual plasmolysis spaces. Scale bars = 50 μm. [file 12870_2020_2501_MOESM6_ESM.tif]
